# Enhancing AI-Driven Medical Translations: Considerations for Language Concordance

**DOI:** 10.2196/70420

**Published:** 2025-04-11

**Authors:** Stephanie Quon, Sarah Zhou

**Affiliations:** 1 Faculty of Medicine, University of British Columbia, 2194 Health Sciences Mall, Vancouver, BC, V6T 1Z3, Canada, 1 6047292089; 2 Faculty of Science, University of British Columbia, Vancouver, BC, Canada

**Keywords:** letter to the editor, ChatGPT, AI, artificial intelligence, language, translation, health care disparity, natural language model, survey, patient education, accessibility, preference, human language, communication, language-concordant care

We commend the recent publication by Dzuali et al [1], which explored the application of ChatGPT for translating patient education materials into multiple languages. This important study highlights a critical area where artificial intelligence (AI) can potentially bridge gaps in language-concordant care. To further this research, we would like to raise several points to enrich the discussion and understanding of the findings.

The study demonstrates that while ChatGPT provides clinically usable translations for Spanish and Russian, its performance with Mandarin is suboptimal. This inconsistency raises important questions regarding the linguistic complexities and structural differences between English and Mandarin, which may hinder the accuracy and appropriateness of translations. Previous research has shown that the nuanced sentence structures and specialized terminology in Mandarin pose challenges for AI models such as ChatGPT, suggesting the need for more refined approaches when using AI for translation in linguistically distinct languages [2].

Being familiar with the Mandarin language, we have firsthand experience with the challenges that come with translating between languages with distinct linguistic structures. Mandarin, with its nuanced sentence structures and specialized terminology, presents difficulties for large language models such as ChatGPT. These challenges are compounded by differences in grammar, idiomatic expressions, and cultural contexts, which may lead to inaccuracies and misunderstandings in translations. Therefore, this study could provide additional insight into how cultural context influences translation quality. Mandarin, for example, involves not only linguistic precision but also an understanding of cultural nuances that could affect comprehension [3]. Future studies could explore how AI models such as ChatGPT are trained to account for these contextual factors to ensure culturally appropriate translations.

Another area for potential exploration in this study is the testing of alternative prompts and the impact they may have on translation quality. While the study focuses on a single translation prompt—“Translate this into <target language>”—the variability of AI-generated translations could be better evaluated through a variety of prompts. Utilizing multiple prompts could reveal a broader range of performance outcomes, especially for linguistically complex languages such as Mandarin and Russian. Other studies have shown that different AI prompts can produce vastly different results [4].

Lastly, the study heavily relies on the involvement of board-certified dermatologists for posttranslation review, which is applicable to the context of dermatology-related information, but may not fully address the extent of errors and misinformation. While human oversight is essential, the study could benefit from a more robust evaluation of how different levels of human intervention—such as linguistic experts or specialists in medical translation—might improve translation accuracy [5]. Future research should explore how different combinations of AI-generated translations and human review from varied sources could optimize clinical usability.

Overall, while ChatGPT shows promise for improving access to language-concordant patient education, further refinement and validation are required. This study is an important milestone in starting this discussion surrounding AI-translation in medical contexts, and we commend the authors for their valuable contribution to advancing the field. They clearly demonstrate a meticulous approach, thoughtful analysis, and commitment to improving patient care through innovative solutions.

## Abbreviations

AI: artificial intelligence
